# Current and Emerging Technologies for Continuous Intraocular Pressure Monitoring in the Control of Glaucoma Progression: A Scoping Review

**DOI:** 10.3390/jcm14248795

**Published:** 2025-12-12

**Authors:** Daniel Monsálvez-Romín, Noelia Martínez-Albert, Mari Carmen García-Domene, Susana Ortí-Navarro

**Affiliations:** Department of Optics and Optometry and Vision Sciences, University of Valencia, C/Dr Moliner 50, 46100 Burjassot, Spain; daniel.monsalvez@uv.es (D.M.-R.); noelia.martinez@uv.es (N.M.-A.); m.carmen.garcia-domene@uv.es (M.C.G.-D.)

**Keywords:** intraocular pressure, continuous monitoring, glaucoma progression, wearable biosensors, implantable biosensors

## Abstract

**Background/Objectives:** Glaucoma affects over 70 million people worldwide and is a major cause of irreversible blindness, with elevated intraocular pressure (IOP) as the only modifiable risk factor. Conventional techniques like Goldmann applanation tonometry (GAT) are widely used but cannot provide continuous or nocturnal monitoring, limiting the detection of pressure peaks relevant to disease progression. Emerging technologies, including home-based devices, wearable sensors, such as contact lens-based sensors (CLBS), and implantable biomedical microelectromechanical systems (bioMEMS), offer more comprehensive and continuous assessment of IOP patterns. Thus, this scoping review aimed to map the available evidence on technologies for continuous IOP monitoring, summarizing their performance and agreement with traditional tonometry. **Methods**: A systematic search of electronic databases was conducted to identify studies published in the last 10 years evaluating self-tonometry devices, CLBS, or implantable systems designed for continuous IOP monitoring. Two reviewers independently screened articles, applied eligibility criteria, charted relevant data, including device characteristics and agreement with GAT, and reported clinical applications. **Results**: Self-tonometry devices demonstrated generally good agreement with GAT while enabling patients to monitor IOP outside clinical settings. These devices provided valuable information on diurnal and nocturnal IOP fluctuations, especially in individuals with rapid progression or those undergoing postoperative follow-up. BioMEMS-based wearable and implantable sensors showed promise for continuous long-term monitoring and revealed previously unrecognized fluctuation patterns, including activity-related changes. **Conclusions**: Emerging IOP-monitoring technologies appear to complement standard clinical methods by offering more detailed IOP profiles. Their integration into clinical practice may support individualized risk assessment and improved management of glaucoma progression.

## 1. Introduction

Glaucoma represents a complex group of ocular diseases unified by a characteristic optic neuropathy that leads to the progressive and irreversible loss of visual function if left untreated, which ultimately results in characteristic and debilitating visual field (VF) defects [1]. This condition poses a significant global health challenge, affecting more than 70 million individuals worldwide. Critically, approximately 10% of those affected experience bilateral blindness, establishing glaucoma as the primary cause of irreversible blindness on a global scale. A major complicating factor in its management is that glaucoma can remain entirely asymptomatic until its advanced stages, when significant and permanent visual damage has already occurred. This latency contributes to a low rate of diagnosis, with estimates suggesting that only 10% to 50% of people living with glaucoma are aware of their condition [2].

Several risk factors are associated with the development of glaucoma. These include advanced age, a family history of the disease (which elevates an individual’s risk by a factor of 2.1), high myopia, and ethnicity [2,3]. However, the most significant and clinically central risk factor is elevated intraocular pressure (IOP), generally defined as a pressure exceeding 21 mm of mercury (mmHg). An increase in IOP can inflict substantial damage upon the ocular structures [4]. Thus, since IOP is the only risk factor currently modifiable, the primary target for all therapeutic interventions is to reduce it [5]. Reducing an elevated IOP (in the 21–32 mmHg range) by 22.5% can decrease the five-year risk of developing open-angle glaucoma (POAG) from 9.5% to 4.4% [6]. Since treatments aim to halt or slow disease progression rather than reverse existing damage, early diagnosis is crucial to minimize any irreversible damage.

Furthermore, IOP is now known to fluctuate throughout the 24 h cycle, with many individuals demonstrating a peak in the early morning or upon waking [7,8,9]. In glaucoma, this 24 h profile often differs from that of healthy eyes, with greater overall fluctuation, a shift in the peak from morning to nighttime hours, or even the absence of a clear peak [8,9,10,11]. Historically, IOP has been assessed using various tonometric techniques, including applanation, rebound, and indentation tonometry, which provide static, point-in-time measurements [12]. However, documenting these diurnal variations with conventional tonometers would require multiple measurements at different times, a process that is both tedious and impractical in a clinical setting.

The growing recognition that IOP fluctuations and nocturnal peaks may play a critical role in glaucomatous progression has highlighted the urgent need for new technologies that enable continuous IOP monitoring, thereby providing a more comprehensive understanding of a patient’s pressure profile to better guide ophthalmological management [13]. Mapping the existing evidence on these technologies is essential to clarify their capabilities, limitations, and potential clinical roles. Therefore, this scoping review aimed to identify and summarize current and emerging technologies designed for continuous and self-administered IOP monitoring and describe their measurement characteristics. Specifically, we sought to (1) identify device types and their measurement characteristics, (2) describe their level of agreement with conventional tonometry, (3) explore their reported clinical applications, including their usefulness in detecting IOP fluctuations relevant to glaucoma progression, and (4) highlight evidence gaps to inform future research and technological development.

## 2. Material and Methods

The present work was conducted through a comprehensive systematic literature review examining current and emerging techniques in IOP monitoring for glaucoma management. The review was conducted following the PRISMA-ScR (Preferred Reporting Items for Systematic Reviews and Meta-Analyses extension for Scoping Reviews) guidelines to ensure systematic rigor and transparency in the identification, screening, and analysis of relevant literature [14,15]. A review protocol was developed to guide study selection, data charting, and synthesis; however, no formal registration was performed. The search was performed across multiple electronic databases, including PubMed/MedLine, Scopus, and Web of Science, covering all records up to 15 October 2025. The search terms were constructed using Boolean operators (AND, OR, NOT) and included combinations of keywords such as: “intraocular pressure”, “continuous monitoring”, “glaucoma progression”, “wearable biosensors”, “implantable biosensors”.

The complete electronic search strategy for all databases was the application of the following search equations:

- “intraocular pressure” AND “glaucoma diagnosis” AND (“control” OR “measurement” OR “monitoring”)
- “intraocular pressure” AND “glaucoma diagnosis” AND (“control” OR “measurement” OR “monitoring” OR “assessment”) AND “devices”
- (“intraocular pressure”) AND (“control” OR “monitoring” OR “assessment” OR “measurement”) AND (“implantable” OR “wearable”)
- (“intraocular pressure”) AND (“control” OR “monitoring” OR “assessment” OR “measurement”) AND (“contact lenses” OR “contact lens”)
- (“intraocular pressure”) AND (“control” OR “monitoring” OR “assessment” OR “measurement”) AND (“biosensor” OR “biosensors”)
- (“intraocular pressure”) AND (“control” OR “monitoring” OR “assessment” OR “measurement”) AND (“eyemate”)
- (“intraocular pressure”) AND (“control” OR “monitoring” OR “assessment” OR “measurement”) AND (“new technologies” OR “new devices” OR “new methods”)
- (“intraocular pressure”) AND (“control” OR “monitoring” OR “assessment” OR “measurement”) AND (“invasive” OR “non-invasive”) AND (“device” OR “devices”)

Furthermore, inclusion and exclusion criteria were defined to ensure the selection of studies most relevant to the objectives and scope of this review.

**Inclusion criteria:**

- Studies published in the last 10 years (2015–2025) to ensure the inclusion of recent advancements in technology.
- Studies related to IOP measurement using emerging technologies.
- Articles published in peer-reviewed journals.
- Studies providing clinical data, technological evaluations, or user feedback on the effectiveness of IOP monitoring devices.
- Research conducted on human subjects or validated through clinical trials.

**Exclusion criteria:**

- Studies published prior to 2015, unless they were significant works or highly relevant.
- Studies focusing on traditional tonometry methods without innovative technological aspects.
- Articles that are reviews, editorials, or conference abstracts without primary or full-text data.
- Duplicate publications or studies without sufficient methodological details.

**Screening and selection process**

The screening process was conducted in different stages:

- Stage 1: Titles and abstracts were screened for relevance to the topic by two independent reviewers. Cohen’s kappa statistic (κ) was employed to evaluate the agreement between the reviewers.
- Stage 2: Full-text articles were assessed for eligibility based on the inclusion and exclusion criteria.
- Stage 3: Relevant data were extracted from eligible studies, including study design, sample size, technology used, outcomes, and limitations.

Two reviewers independently extracted data from all included studies. Cohen’s kappa statistics assess inter-rater reliability by quantifying the degree of agreement between reviewers while accounting for the possibility of chance agreement. The kappa value ranges from −1 to 1, with values closer to 1 indicating higher levels of agreement. The calculated Cohen’s kappa statistic for this study was κ = 0.87, indicating almost perfect agreement.

**Figure 1:**
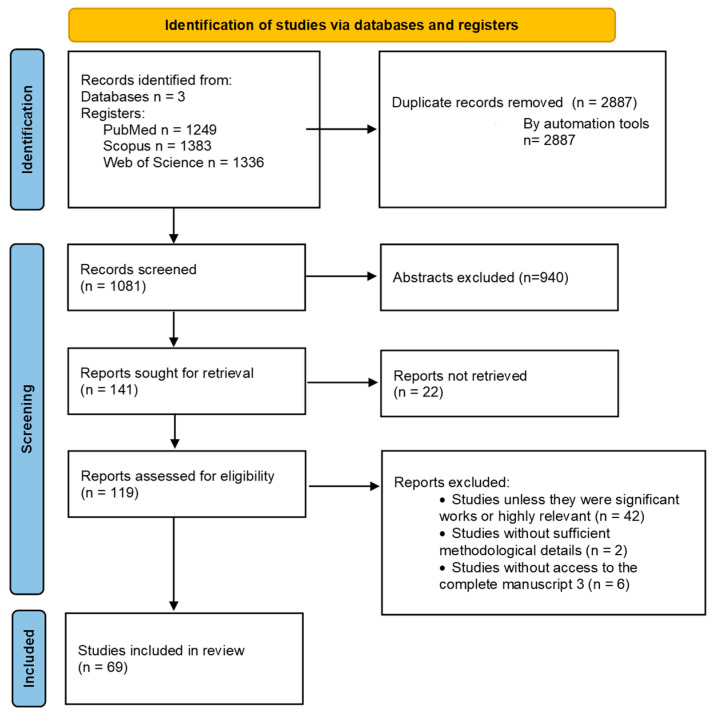
Flow diagram of Scope Review.

Extracted data were organized according to study characteristics, population, technology used, outcomes, and reported limitations. Data were summarized descriptively using tables to map key features across studies. The study selection process is illustrated using the PRISMA flow diagram (Figure 1), which provides a transparent overview of the number of records identified, screened, excluded, and included in this review.

## 3. Results

A total of 1081 records were identified through databases. After removal of duplicates and screening of titles and abstracts against the inclusion criteria, full texts of potentially relevant articles were assessed for eligibility. Following full-text review, 69 articles met all criteria and were included in this scoping review.

The 69 included studies encompassed a variety of designs, including clinical studies, technical evaluations, comparative analyses, and discussion or review papers on emerging technologies. Key characteristics charted from each study included study design, sample size, population, technology evaluated, reported outcomes, and limitations. These characteristics are summarized in Table 1, Table 2 and Table 3, with full citations provided.

## 4. Discussion

### 4.1. Self-Tonometry Devices

Self-tonometry devices such as the iCare HOME (ICH, FDA-approved in 2017) and iCare HOME 2 (ICH2, FDA-approved in 2022) are increasingly important in glaucoma management, enabling patients to monitor IOP outside clinical settings. By allowing multiple daily measurements, they provide a more detailed picture of diurnal IOP fluctuations, overcoming the limitations of traditional clinic-based testing. The ICH and the ICH2 use rebound tonometry, which measures the deceleration of a magnetized probe rebounding from the cornea, without the need for anesthetic. Readings are taken 4–8 mm from the eye, with valid results based on an average of four out of six measurements [44]. The ICH2 introduces key improvements, including an onboard display, adjustable facial supports, LED alignment guide, and a reliability score for quality assessment [19]. Both devices integrate with mobile apps, allowing efficient data storage and remote sharing with healthcare professionals for enhanced glaucoma monitoring.

#### Evaluation and Agreement with Gold-Standard

Several recent studies have examined these devices to determine their feasibility, user acceptance, measurement consistency, and agreement with the gold-standard GAT method (Table 1).

Assessment of the original ICH (model TA022) compared to GAT has yielded detailed findings regarding clinical concordance. Nayak et al. [16], comparing the ICH and GAT in both normal and diagnosed glaucoma patients (primarily using the right eye), reported a strong overall agreement rate of 94.7%, with a mean difference of 0.83 mmHg. Specifically, in normal subjects, the mean IOP values were 12.4 ± 3.5 mmHg with ICH and 13.4 ± 3.2 mmHg with GAT. For the glaucomatous group, the mean values were 23.6 ± 11.9 mmHg (ICH) and 24.2 ± 11 mmHg (GAT). This study demonstrated that the ICH maintains good clinical concordance (agreement above 90%) across various pressure levels, though range-dependent bias was observed. For the low IOP range (7–16 mmHg), the ICH generally underestimated the IOP compared to GAT (mean difference 1.22 mmHg, 97.4% agreement). In the middle IOP range (17–23 mmHg), the mean difference decreased slightly to 0.77 mmHg (91.3% agreement). Conversely, in the higher IOP range (>23 mmHg), the ICH exhibited a slight overestimation of the IOP compared to GAT (mean difference −0.11 mmHg, 100% agreement). Only for higher values (IOP > 23 mmHg), the IOP with ICH was slightly overestimated (mean difference −0.11 mmHg).

Conversely, a study by Kadambi et al. [17] found the agreement to be more limited in glaucoma patients and suspects, reporting a wider mean difference of 2.2 mmHg and wider 95% limits of agreement (LoA) (−5.7 to 10.1 mmHg). Overall, ICH mean IOP (as determined by an experienced optometrist) was within 2 mmHg of GAT IOP in 39.2% (40/102 eyes), within 3 mmHg in 56.86% (58/102 eyes), and within 5 mmHg in 77.84% (79/102 eyes). Additionally, the difference between ICH and GAT measurements was found to be significantly affected by CCT, with ICH overestimating IOP compared to GAT as CCT increased. Despite varying concordance outcomes, the ICH consistently demonstrated good repeatability results, achieving an ICC of 0.957.

The newer ICH2 version has also shown promising clinical agreement with GAT. Quérat and Chen [18] conducted a prospective pilot study comparing unsupervised ICH2 recordings by glaucoma participants to GAT. They found good agreement, noting mean IOP values in the right eye were 19.46 ± 7.09 mmHg (ICH2) and 19.63 ± 6.35 mmHg (GAT), resulting in an ICC of 0.907. For the left eye, means were 16.96 ± 5.18 mmHg (ICH2) and 17.04 ± 4.61 mmHg (GAT), with an ICC of 0.830. However, regression analysis revealed that the difference between GAT and ICH2 measurements increased progressively when the IOP exceeded 20 mmHg for the right eye and 17 mmHg for the left eye. Furthermore, the standard deviation of the measurements with the ICH2 was slightly higher, indicating greater variability in the readings, likely due to the home-use conditions.

An observational cross-sectional study by Romano et al. [19] further supported these findings, reporting a small mean difference between GAT and ICH2 of −0.28 ± 1.57 mmHg (95% LoA: −3.36 to –2.79 mmHg). ICH2 also demonstrated good repeatability, with a mean difference between the first and second measurements of −0.23 ± 1.04 mmHg, (95% LoA: −2.27 to 1.81 mmHg). Nonetheless, this study also noted that ICH2 overestimated high IOPs and underestimated low IOPs relative to GAT, and that thicker corneas led to proportionally higher rebound tonometer readings.

In addition to clinical accuracy, patient satisfaction and comfort are crucial for the successful long-term adoption of self-tonometry devices. Overall, patient impressions and satisfaction with both the ICH and ICH2 have been overwhelmingly positive, demonstrating high feasibility and acceptability among motivated patient cohorts. In a prospective study, Hu et al. [44] reported that 73.7% of participants found the ICH tonometer easy to use, and all (100%) considered it useful. Furthermore, 84.2% indicated they would continue using it in future telehealth appointments. After receiving proper instruction, all participants felt confident in operating the device independently, and 98.6% of the 16 measurements per eye were deemed acceptable. Despite this, 44% expressed a preference for GAT over ICH. High satisfaction persisted with the newer ICH2 model. Quérat and Chen [18] found that 92% of users were satisfied with the instructional film and 88% with the supporting smartphone application. However, 36% would have preferred hands-on guidance during training.

Qualitative feedback from Hu et al. [44] and Quérat and Chen [18] emphasized the advantages of home monitoring, including convenience, accessibility, a sense of empowerment, and improved confidence in managing chronic conditions. Patients also viewed rebound tonometry as safer and more comfortable than GAT, as it avoids anesthetic drops and corneal staining. Nayak et al. [16] reported that only 3.9% of participants experienced moderate discomfort (Grade 2). The main challenge noted was difficulty with alignment, identified by Kadambi et al. [17] as the most frequent issue (43% of participants).

### 4.2. Biosensors

Advances in microelectronics have enabled the development of bioMEMS (biomedical microelectromechanical systems) for continuous intraocular pressure (IOP) monitoring, essential in glaucoma management [45,46]. These devices are classified as implantable or wearable biosensors. A biosensor consists of a bioreceptor, which identifies a specific target, and a transducer, which converts this interaction into a measurable signal [7]. For effective IOP sensing, devices must be safe, biocompatible, durable, and capable of reproducing clinical measurement accuracy [46].

Current technologies include wearable contact-lens-based sensors (CLBS) and implantable pressure-sensing bioMEMS. CLBS offer a minimally invasive approach by detecting pressure changes on the ocular surface, while implantable sensors provide direct and highly accurate intraocular measurements. Both types allow continuous tracking of IOP fluctuations, offering valuable data to personalize treatments and improve outcomes [7,8].

The biosensors within these devices use pressure transducers with diverse sensing mechanisms. On the one hand, the piezoresistive sensors measure resistance change due to strain/pressure. On the other hand, capacitive sensors detect capacitance change from diaphragm deflection. Other types include optical devices, which use light interference, whereas microfluidic sensors measure the fluid movement response to the IOP [46,47,48]. These devices use advanced materials like polymers, nanomaterials, or silicon-based substrates [49,50,51].

The following subsections will provide insights and current findings regarding these various systems for the measurement of IOP.

### 4.3. Contact-Lens-Based Sensor Wearable Devices

Several authors have evaluated the results obtained with the use of CLBS in humans (Table 2).

There are various sensors for obtaining IOP values with CLBS. However, they are all based on the principle that rising IOP induces changes in corneal curvature which is used to detect subtle IOP fluctuations and provide immediate feedback to patients and physicians [52,53]. The SENSIMED Triggerfish^®^ (Sensimed S.A., Etagnières, Switzerland) has been used in most human studies. Nevertheless, this device does not provide IOP measurements in mmHg, but rather an electrical voltage equivalent (mVEq), which is its main limitation [20]. However, novel sensor systems that can output IOP continuously in mmHg are under current research [23,28].

#### Evaluation and Agreement with Gold-Standard and Other Methods

Regarding the assessment of the agreement, Wasilewicz et al. [21] evaluated a new CLBS for continuous measurements of IOP and ocular pulse amplitude (OPA) over 24 h. The mean IOP difference between CLBS and tonometry in the same eye was ±5 mmHg in 75% (GAT) and 87.5% of subjects with dynamic contour tonometry (DCT). IOP readings obtained by CLBS were generally higher than GAT measurements (on average 2.75 mmHg higher) but close to DCT values (on average 0.18 mmHg higher). Similarly, Gillmann et al. [22] developed a CLBS that continuously measures IOP in mmHg. The mean difference in successive IOP measurements performed with pneumatonometry and CLBS was 2.0 ± 4.3 mmHg at the time of insertion and 6.5 ± 15.2 mmHg at the time of removal. These results show acceptable accuracy in measuring IOP values compared to pneumatonometry, as well as good sensitivity to subtle IOP variations.

Another study by Beltrán-Agulló et al. [29] determined the differences in the data provided by the SENSIMED Triggerfish CLBS and GAT. They observed a significant increase in IOP measurements with the GAT (*p* = 0.001) and in measurements after 24 h with the CLBS (*p* = 0.02) (drift phenomenon). More recently, Wei et al. [20] evaluated the accuracy of a new CLBS, and measured GAT IOP in the sitting position, and with the Perkins Applanation Tonometer (PAT) in the supine positions. No significant differences were found between pre-CLBS GAT and post-CLBS GAT in normal eyes or between all comparisons in POAG/OHT eyes with high IOP (*p* > 0.5). Furthermore, the intraclass correlation coefficient was observed to be moderate to very consistent (0.51 ≤ r ≤ 0.95; *p* < 0.05). Bland–Altman analysis showed that more than 80% of the points were within ±5 mmHg and more than 60% were within ±3 mmHg.

When using devices that interact with the ocular surface, patient comfort, satisfaction, and potential adverse effects are key considerations. CLBS have been evaluated in several studies for these outcomes. Otsuka et al. [36] assessed 56 patients using a CLBS and found blurred vision in 55%, eye pain in 30%, conjunctival hyperemia in 14%, and sleep disturbances in 29%, with blurred vision more common in those with good visual acuity. Zhang et al. [35] evaluated a novel CLBS, monitoring corneal fluorescein staining (CFS), ocular surface disease index (OSDI), tear break-up time (TBUT), best-corrected visual acuity (BCVA), and subjective discomfort before, immediately after, and one day post-measurement. They observed transient increases in CFS, OSDI, and discomfort, and temporary reductions in TBUT and BCVA, all returning to baseline within a day. Wasilewicz et al. [21] reported corneal erosions resolved in 3.1 ± 1.8 days with or without treatment. Huang et al. [32] noted no serious adverse events, though transient blurred vision and conjunctival hyperemia were common. Carnero et al. [30] also observed mild to severe hyperemia, which resolved within a day. Overall, CLBS devices are generally safe and well-tolerated, but patients should be informed about possible transient visual disturbances, eye discomfort, and mild ocular surface changes.

### 4.4. Intraocular Implantable bioMEMS

In recent years, novel intraocular bioMEMS have emerged that enable continuous monitoring of IOP from within various ocular structures, and these more invasive technologies are currently being evaluated (Table 3).

A key device in clinical use is the EYEMATE-IO (Implandata Ophthalmic Products GmbH), which incorporates pressure-sensitive capacitors, received its latest CE certification in 2021, and was initially tested under the name ARGOS [38]. This system is designed to be implanted within the ciliary sulcus, a strategic placement that facilitates concurrent insertion during routine cataract surgery, integrating it into the standard management workflow. In contrast, the EYEMATE-SC is a miniaturized variant intended for patients who are not undergoing cataract surgery, utilizing an alternative configuration that positions it within the suprachoroidal space for standalone glaucoma procedures without the need for concurrent cataract extraction. To obtain measurements from these devices, a reader unit is typically required, which must be held at a short distance in front of the eye [46].

#### Evaluation and Agreement with Gold-Standard

The EYEMATE-IO sensor system, assessed across multiple trials, has established itself as a generally safe and reliable technology for long-term, continual IOP monitoring in glaucoma patients [38,39,40]. The long-term follow-up study by Koutsonas et al. [38] evaluated the first-generation EYEMATE-IO in patients with POAG and demonstrated the device’s good functionality and tolerability over an average period of 37.5 months. This device successfully provided extensive data via home self-tonometry, averaging 1273 readings per patient. Follow-up at three years confirmed stable ocular findings and continued patient comfort. However, this initial generation showed significant limitations, including mechanical stress effects like pupillary distortion in all patients and iris atrophy in one case, as well as frequent measurement shifts needing recalibration and a consistently poor correlation with GAT.

The limitations of the first-generation device led to the development of a smaller, redesigned second-generation EYEMATE-IO, evaluated by Choritz et al. [39] in patients with POAG. Over 12 months, the new sensor showed safe implantation, reliable performance, and no unexpected adverse events. The redesign improved biocompatibility, reducing inflammation and pigment dispersion, and demonstrated good agreement with GAT (ICC = 0.783). However, the sensor readings were systematically higher by a mean difference of 3.2 mmHg (95% LoA: −3.8 to 10.2 mmHg), showing an increasing difference between EYEMATE-IO and GAT at higher IOPs. Patients used the device frequently (7.9 ± 1.4 measurements per day), confirming its suitability for continuous IOP monitoring and detection of diurnal pressure patterns.

Perhaps the most compelling result for the EYEMATE-IO system relates to its clinical relevance in predicting structural progression. A recent publication by Micheletti et al. [40] evaluated the association between continuous IOP data gathered by the implanted EYEMATE-IO sensor and the concurrent rates of retinal nerve fiber layer (RNFL) thinning in patients with POAG. The main findings established that both peak IOP and IOP fluctuations (defined as the SD of measurements) were significantly associated with faster RNFL thinning. IOP fluctuation demonstrated the strongest correlation, as a 1-mmHg increase in SD of IOP was associated with a 0.76 μm/y faster RNFL thinning rate. In contrast, the routine in-office mean IOP measured by GAT showed no statistically significant association with the rates of RNFL thinning over the same period.

In parallel, the EYEMATE-SC sensor has been evaluated in several studies [41,42,43] demonstrating its long-term safety, tolerability, and reliability for continuous IOP monitoring. The initial 6-month report by Szurman et al. [41] found the sensor was successfully implanted in all 24 eyes with POAG, reporting no device migration, dislocation, or serious device-related complications during the follow-up. The most frequent complication observed was surgery-related hyphema (in nine eyes), which resolved spontaneously. Performance comparison showed an overall mean difference of 1.31 mmHg between GAT and EYEMATE-SC (95% LoA: from −4.92 to 7.55 mmHg), but this agreement significantly improved over time. By 6 months, the mean difference reached a close agreement of −0.15 ± 2.28 mmHg, and 100% of measurements were within ±5 mmHg of GAT. The subsequent 12-month evaluation of the EYEMATE-SC sensor by Szurman et al. [42] provided extended confirmation of the safety and stability of the device, recording no migration, rotation, dislocation, or serious device-related complications. The overall mean difference between GAT and EYEMATE-SC measurements across 536 comparisons was 0.8 mmHg (95% LoA: −5.1 to 6.7 mmHg). Crucially, the accuracy stabilized after the initial postoperative phase (3 months), showing a consistent and improved agreement for the remaining follow-up, with a mean difference in only −0.2 mmHg from 3 months onward. This stable agreement meant that 100% of measurements taken at 6, 9, and 12 months were within ±5 mmHg of GAT. Recently, a long-term follow-up study by Micheletti et al. [43] over a mean period of 2.7 ± 0.6 years further confirmed the long-term safety and tolerability of the EYEMATE-SC system, documenting no serious Adverse Events (AEs) or device-related AEs (ADEs) and no suprachoroidal migration. Performance analysis comparing the EYEMATE-SC to GAT showed good agreement, with overall mean differences in −0.3 mmHg and 95% LoA ranging from −6.2 to 5.7 mmHg. Importantly, 94% of all paired measurements collected during the 2-year follow-up were recorded within ±5 mmHg of GAT.

In summary, this review aimed to analyze the results of current and emerging technologies for the continuous monitoring of IOP with up-to-date clinical assessments. The landscape of IOP monitoring technologies presents a critical trade-off between device invasiveness, resulting data quality, and feasibility for widespread clinical application. Current and emerging devices fall into three categories: non-invasive self-tonometry (ICH/ICH2), minimally invasive wearable biosensors (CLBS), and highly invasive intraocular implantable bioMEMS. While all aim to overcome the fundamental limitation of traditional GAT, missing critical diurnal IOP fluctuations, their adoption is governed by distinct strengths and operational limitations.

### 4.5. Comparative Utility and Measurement Reliability

Self-tonometry devices, such as the iCare HOME (ICH) and ICH2, excel in accessibility and patient acceptance, offering a non-invasive solution that empowers patients with continuous monitoring in a home setting. The use of rebound tonometry is generally perceived as safer and more comfortable than GAT, as it avoids anesthetic drops and corneal staining. The ICH/ICH2 generally demonstrates good clinical concordance and repeatability compared to GAT across multiple studies. However, the agreement was not always deemed clinically interchangeable due to wide LoA [17]. A recurring finding was an IOP-dependent bias, where the rebound tonometers tended to slightly underestimate IOP in the lower ranges and slightly overestimate in the higher ranges. Additionally, the difference between ICH and GAT was significantly affected by CCT, with the ICH device tending to overestimate IOP as CCT increased. These findings support the use of these self-tonometers as a viable option for patients to track their IOP at home. However, prior instruction by trained professionals is required to ensure correct handling and interpretation. The use of these devices is subject to some limitations. Patient selection criteria commonly exclude individuals with high astigmatism (e.g., >3 diopters), as well as those with corneal scars, microphthalmos, nystagmus, keratoconus, or other corneal or conjunctival pathologies or infections, and those with recent cataract extraction [44]. Adequate fixation is essential, as eccentric fixation is not acceptable [16]. Patients should have good corneal health and a CCT within normal limits since measurements may be influenced by CCT, and also by corneal hysteresis and the corneal resistance factor, while remaining independent of corneal curvature [9,16].

Wearable biosensors, primarily CLBS, offer the crucial strength of continuous, high-resolution tracking of IOP fluctuations, which are often undetectable by single in-office GAT measurements [24]. Data gathered by CLBS, such as 24 h IOP fluctuations and ocular pulse amplitude, are considered valuable risk factors for VF and may help adjust treatment strategies earlier. Thus, subjects previously classified as normal-tension glaucoma patients can be diagnosed as POAG when diurnal IOP peaks captured by CLBS are taken into account, improving diagnostic accuracy [31,33,34]. Gaboriau et al. [25] conducted a cross-sectional study using CLBS and determined that 24 h IOP fluctuations may act as a risk factor for VF progression, and that the CLBS may help adjust treatment strategies earlier. Previously, Hoban et al. [54] analyzed the relationship between glaucomatous VF and the amplitude of a 24 h IOP-related profile measured by a CLBS and concluded that new parameters obtained using CLBS could provide information to predict the risk of VF changes in glaucoma patients. The study by De Moraes et al. [26], with a larger sample size, reported that ocular pulse amplitude (measured by a CLBS) and baseline mean deviation were associated with VF progression. Tojo et al. [27] also studied the correlation between IOP monitoring with CLBS and VF in 69 patients and showed that high peak amplitudes during the 24 h period, as well as during the nocturnal period, were associated with an increased risk of VF progression in glaucoma patients. In addition, several studies have reported variations in mean values depending on body posture and head tilt [20,27,28,29].

Agreement between CLBS outputs and standard tonometric measurements remains inconsistent. Objective safety assessments indicate that CLBS use can induce transient increases in corneal fluorescein staining and reductions in TBUT. Although topographic changes have been observed in both healthy and glaucomatous eyes following CLBS wear, corneal biomechanical properties appear largely unaffected [37]. A key limitation of widely studied systems such as the SENSIMED Triggerfish is their inability to provide direct IOP measurements in mmHg. The existing evidence base is further constrained by limited cohort homogeneity (sex and age distribution) and small sample size, issues that are likely to persist until these technologies are more fully characterized and technically optimized. Ongoing investigations are evaluating the performance of various CLBS across multiple animal models to validate their ability to detect physiologically meaningful IOP fluctuations with accuracy, safety, and reliability [55,56,57,58,59,60,61,62,63,64,65,66,67,68,69,70]. Additional work using CLBS in silicone eye models also highlights their promise for continuous IOP monitoring [71,72,73]. Continued research is needed to ensure long-term stability and biocompatibility, minimize adverse ocular responses, and refine manufacturing processes to achieve consistent and reliable performance of these next-generation materials [49,50].

The highly invasive intraocular implantable bioMEMS provide the most clinically potent data. Their main strength lies in long-term reliability and direct, accurate intraocular measurement, with modern systems achieving stable agreement with GAT measurements over time. Nevertheless, intraocular monitoring devices have notable limitations. In addition, the first-generation device may rotate over time, although this event does not affect its function. The second generation avoids this with the inclusion of haptics in the design [39]. Occasional effects such as pupillary distortion or iris transillumination may occur from intraoperative pigment dispersion, and rare complications include temporary corneal decompensation or uncontrolled IOP spikes requiring glaucoma surgery. Furthermore, these devices may require recalibration at some point [38,39,40].

Several studies have examined external factors that might affect measurements from these devices. Van den Bosch et al. [74] evaluated a subset of patients from the ARGOS-02 study diagnosed with early stage POAG. It was observed that actions such as eyelid squeezing or eye rubbing could produce temporary elevations of more than 60 mmHg relative to baseline measurements. However, in these specific studies, the exact force applied during rubbing cannot be precisely known, and the measurements are punctual, meaning the long-term implication of these habits is not well understood. Other potential influences that have been investigated include electromagnetic radiation emitted by devices such as smartphones or laptops; regarding this, it has been found that there are no fluctuations in the telemetric reader measurements at the frequencies studied [75], given that the devices are tested and certified for electromagnetic and radiation safety. Regarding whether the implantation of intraocular monitoring devices influences disease progression, long-term follow-up studies focusing on the sensor found no evidence of a causal relationship between the sensor or the implantation procedure and the observed progression [38].

### 4.6. Practical Barriers to Clinical Implementation

Clinical adoption of IOP monitoring devices is constrained by device-specific limitations, and not all patients are suitable candidates for wearable or implantable systems. Self-tonometry requires prior training, precise fixation, and excludes patients with corneal abnormalities, making measurements user- and cornea-dependent. CLBS offer continuous monitoring but are associated with ocular surface complications, require strict lens care, and often need hospitalization during testing, limiting real-world data capture. Implantable BioMEMS provide the most accurate and clinically relevant data but are invasive, restricted by eye anatomy, and carry surgical and device-specific risks. As intraocular procedures, implantation carries risks such as hyphema, with eligibility restricted to eyes of specific axial lengths. Specifically, they are not recommended for very short (<22 mm) or very long eyes (>26 mm) due to higher surgical risks and the potential to increase pupillary block [38]. When combined with cataract surgery, use in children and young adults is limited. Device-specific complications including pupillary distortion, iris transillumination, and transient corneal decompensation, necessitate clinical supervision.

In this context, it must be remembered that these devices serve primarily to continuously monitor IOP and, therefore, the disease can continue to progress if it is not addressed therapeutically. Therapeutic approaches include IOP-lowering strategies, sometimes involving intraocular surgery procedures with drug delivery devices for glaucoma treatment or contact lenses with elution capabilities to provide controlled drug release directly to the eye [49,50], although these treatment modalities are outside the scope of this review.

### 4.7. Future Perspectives

Several new devices for continuous IOP monitoring are currently in development. Many of them aim for greater miniaturization of the sensors to minimize ocular side effects and facilitate implantation [47,76,77]. Additional technologies under investigation include an interferometry-based system, which shows promise but has not yet been tested in humans [78]. Emerging substrates such as graphene are also being actively researched, with the expectation that it will be possible to commercialize devices using these materials in the future. Corneal factors, such as biomechanics and thickness, can also influence readings. However, advances in algorithmic processing can partially compensate for these effects [71]. Further research and clinical trials will be needed to evaluate efficacy and safety in real-world applications.

## 5. Conclusions

In conclusion, technological advancement has allowed sophisticated devices to continuously monitor IOP in both healthy and glaucomatous eyes in a precise, secure, and clinically useful manner. However, with many current and novel methods, a complete 24 h monitoring is not always feasible without disrupting the sleep–wake cycle. Different methods exist, each with their advantages and disadvantages, greater or lesser invasiveness, and greater or lesser comparability with gold-standard methods, as reported in this updated review. Therefore, ongoing research is needed to refine the technology and fully establish its clinical benefits and to determine if the increased number of IOP measurements translates to better glaucoma control and therapy. These devices can be extremely useful in further future research. For example, continuous monitoring may be necessary to draw robust conclusions about whether IOP is associated with specific daily activities such as playing a musical instrument or engaging in sports that may induce transient IOP fluctuations and are not captured by sporadic clinic measurements [9]. In this context, these monitoring devices could be particularly useful for identifying activity-related IOP patterns and previously undescribed risk factors, thereby informing individualized risk assessment and management strategies in the control of glaucoma progression. The limitations of our review include the lack of research to which we did not have access and other sources of unpublished studies or studies with insufficient information or those in the process of commercializing the product.

## Figures and Tables

**Table 1:** Summary of Performance of iCare Home tonometers.

Article & Device	Study Design & Population	Agreement Results	Repeatability Results	Key Findings
**Nayak et al., 2023** [16] (iCare HOME)	Prospective observational study. 76 certified Indian patients (out of 83 recruited). MA: 53.1 ± 15.5 years M: 53%-F: 47%	**MD** ICH vs. GAT: 0.83 mmHg (**95% LoA**: −2.25 to 3.92 mmHg), 94.7% agreement. High IOP (>23 mmHg): −0.11 mmHg (**95% LoA**: −2.74 to 2.52 mmHg), 100% agreement.	**Precision (ICC):** 0.996 (95% CI 0.994–0.998). **CoV:** ICH = 6.6; GAT = 3.4.	Patients performed self-tonometry with good reliability and safety, showing good agreement with GAT.
**Kadambi et al., 2023** [17] (iCare HOME)	Prospective observational study. Diurnal Variation Testing. 51 patients (102 eyes) with glaucoma and suspects. MA: 53 ± 16 years M: 63%-F:27%	**MD** ICH vs. GAT: 2.2 mmHg (**95% LoA**: −5.7 to 10.1 mmHg). ICH Optometrist vs. Participant: 0.1 mmHg (95% LoA: −5.3 to 5.5 mmHg).	**Intradevice (ICH):** ICC = 0.95 (95% CI 0.94–0.97). **Interrater (ICH):** ICC = 0.916 (95% CI 0.787–0.960).	Agreement between ICH and GAT was limited; home tonometry cannot substitute GAT DVT.
**Quérat and Chen, 2025** [18] (iCare HOME2)	Prospective observational pilot study. 25 motivated participants with glaucoma or suspects. MA: 61 ± 17 years M: 52%-F: 48%	**MD** ICH2 vs. GAT: RE: 0.17 mmHg. LE: 0.08 mmHg. **Bias**: Difference increased progressively when IOP was above 20 mmHg (RE) or 17 mmHg (LE).	Not reported	Demonstrated feasibility and acceptability of unsupervised self-tonometry with good to excellent agreement.
**Romano et al., 2025** [19] (iCare HOME2)	Observational cross-sectional study. 104 glaucoma patients and glaucoma suspects. MA: 58.2 ± 14.6 years M: 58%-F: 42%	**MD** ICH2 vs. GAT: −0.28 ± 1.57 mmHg (**95% LoA**: −3.36 to 2.79 mmHg). **MD** ICH2 vs. IC200: 0.92 ± 1.48 mmHg (**95% LoA**: −1.98 to 3.82 mmHg).	**LoR**: **GAT**: 95% LoR (−1.71 to 2.13 mmHg). **ICH2**: 95% LoR (−2.27 to 1.81 mmHg). **IC200**: 95% LoR (−2.20 to 2.16 mmHg)	Self-measured IOP showed good reliability and excellent repeatability compared to physician-measured IOP using both GAT and rebound tonometry.

CI: confidence interval; CoV: coefficient of variation; DVT: diurnal variation testing; F: female; GAT: Goldmann applanation tonometry; ICC: Intraclass Correlation Coefficient; ICH: iCare Home; IOP: intraocular pressure; LE: left eye; LoA: limits of agreement; LoR: limits of repeatability; M: male; MA: mean age; MD: mean difference; RE: right eye. Bold text is used to emphasize the articles’ authors and key results.

**Table 2:** Summary of Performance of Contact-lens-based sensor wearable devices.

Article & Device	Study Design & Population	Main Objective	Key Findings
**I. Device Accuracy, Feasibility, and Direct IOP Measurement**			
**Wei et al., 2025** [20] Pressure-Measuring Contact Lens	Cross-sectional study. 80 eyes (40 Normal, 40 POAG/OHT). MA: 23.8 ± 3.2 years (NE). MA: 35.0 ± 14.8 years (POAG). MA: 37.1 ± 9.1 years (OHT). M: 50%-F: 50%.	Evaluate the **accuracy** against GAT in seated and supine positions.	**Good Agreement** with GAT in sitting and supine positions: Mean IOP differences between CLBS and GAT were consistently <2 mmHg in all groups.
**Wasilewicz R et al., 2020** [21] Pressure-Measuring Contact Lens (mmHg)	Prospective, open, single-center, non-randomized study. 8 subjects (4 Healthy, 4 Glaucoma). MA: 52.9 ± 17.2 years. M: 37.5%-F: 62.5%.	Assess **feasibility** for 24 h continuous IOP and OPA measurement.	**Good agreement** with GAT: Mean IOP difference −2.75 ± 3.52 mmHg. The IOP difference within ±5 mmHg in 75% of subjects.
**Gillmann, et al., 2021** [22] Pressure-Measuring Contact Lens (mmHg)	Prospective study. 8 subjects (4 Healthy, 4 POAG/NTG). MA: 63.0 ± 18.2 years (NE) MA: 42.7 ± 9.4 years (POAG). M: 25%-F: 75% (NE). M: 50%-F: 50% (POAG).	Assess **reliability/accuracy** against pneumatonometry, validating ability to measure IOP in mmHg.	**Fair Accuracy and Sensitivity**: Confirmed capability for IOP values measurement in mmHg and good sensitivity to subtle IOP variations. **Good agreement**: 88.0% of IOP variations measured by CLBS were within ±5 mmHg of pneumatonometry in the fellow eye over 24 h.
**II. Nyctohemeral Patterns, Variation, and Risk Factors**			
**Zhang et al., 2024** [23] Pressure-Measuring Contact Lens	Descriptive analysis. 59 normal Chinese adults. 2 groups: G1 > 30 and G2 < 30 years. M: 37.3%-F: 62.7%.	Investigate **physiological nyctohemeral rhythms** in normal adults.	**Stable Mean IOP:** 24 h mean IOP was comparable between day and night (*p* = 0.695). **Demographics Impact:** Subjects ≥ 30 years old had higher 24-h mean IOP and larger MAPE.
**Kim et al., 2020** [24] SENSIMED Triggerfish (mVEq)	Prospective case–control study. 30 NTG eyes, 20 Healthy controls. MA: 65.2 ± 7.7 years (NE). MA: 57.7 ± 10.1 years (NTG). M: 40%-F: 60% (NE). M: 46.7%-F: 53.3% (NTG).	Investigate 24 h IOP-related **patterns and association with NTG risk**.	**Greater Variation in NTG**: NTG eyes showed greater 24 h variation. **Nocturnal Peak:** CLBS measurement values during the nocturnal acrophase were significantly greater in NTG than in healthy controls.
**Gaboriau et al., 2023** [25] SENSIMED Triggerfish (mVEq)	Cross-sectional prospective study. 54 POAG patients (Fast vs. Slow progression groups). MA: 69.6 ± 6.2 years. M: 50%-F: 50% (G1). M: 63.6%-F: 36.4% (G2).	Compare IOP fluctuations between POAG patients with **different VF progression rates**.	**CCT and Progression:** Patients with a faster progression rate (Mean deviation rate < −0.5 dB/year) had significantly thinner mean CCT.
**De Moraes et al., 2018** [26] SENSIMED Triggerfish (mVEq)	Multicenter retrospective cohort study. 445 eyes (Treated POAG). MA: 68.9 ± 11.2 years. M: 46.5%-F: 53.5%.	Test if 24 h CLBS patterns correlate with **prior rates of VF progression.**	**CLBS Correlates with Progression:** A single 24 h CLBS recording provided a signature associated with prior rates of VF progression.
**Tojo et al., 2020** [27] SENSIMED Triggerfish (mVEq)	Not reported study design. 69 glaucoma eyes (Follow-up > 2 years). MA: 70.8 ± 8.5 years. M: 49.3%-F:50.7%.	Investigate correlations between 24 h CLBS measurement and **VF progression.**	**Fluctuation Predicts Progression:** Large SD of IOP fluctuation during the 24 h, diurnal, and **especially nocturnal periods** (*p* = 0.0027) were identified as risk factors for rapid VF progression.
**III. Positional Effects and Environmental Triggers**			
**Zhang et al., 2025** [28] Pressure-Measuring Contact Lens (mmHg)	Prospective comparative study. 20 Normal, 14 HTG, 16 NTG, 14 OHT (all untreated). MA: 31.1 ± 10.0 years (NE). MA: 29.7 ± 7.5 years (HTG). MA: 38.1 ± 10.6 years (NTG). M-F Not reported.	Evaluate CLBS efficacy in detecting continuous IOP variations following **positional transitions** (sitting, supine, HDT).	**Increased IOP on Change**: Normal subjects, HTG, and NTG patients showed higher CLS IOP mean/peak in supine/HDT compared to sitting (*p* < 0.05). **HTG Hyper-Responsiveness**: HTG showed higher IOP increment and velocity during the sitting-to-supine transition than normal subjects (*p* < 0.05). **OHT Non-Responsiveness**: OHT subjects generally did not show significant IOP differences across the 3 positions (*p* > 0.1).
**Beltran-Agulló et al., 2017** [29] SENSIMED Triggerfish (mVEq)	Prospective, randomized, cross-over, open-label comparative study. 12 subjects with progressive POAG or NTG. MA: 67.9 ± 8.9 years. M: 16.7%-F: 83.3%.	Determine the effect **of sleeping flat versus 30° head-up position** on IOP-related patterns.	**Inconsistent Positional Effect**: No significant difference in mean values was observed between positions (*p* = 0.51).
**Carnero et al., 2020** [30] SENSIMED Triggerfish (mVEq)	Prospective observational study. 19 patients suspected of having OSAS (11 Severe, 8 Non-Severe). MA: 49.3 ± 9.5 years (no severe). MA: 55.9 ± 10.3 years (severe). M: 80%-F: 20%.	Analyze nocturnal **IOP fluctuations and correlation with OSAS severity.**	**Periods of nocturnal IOP elevation** were longer in patients with severe OSAS and correlated with disease severity. Patients with OSAS may have normal IOP measurements during medical visits, but their IOP may be significantly elevated at night.
**IV. Treatment Efficacy and Diagnostic Utility**			
**Posarelli et al., 2019** [31] SENSIMED Triggerfish (mVEq)	Cross-sectional, nonrandomized, prospective, pilot study. 89 POAG (G1: Ex-PRESS mini glaucoma; G2: Hydrus microstent implantation; G3: treated medically). MA: 71.1 ± 11.4 years (G1). MA: 77.8 ± 4.6 years (G2). MA: 73.3 ± 4.8 years (G3). M: 58.6%-F: 41.4% (G1). M: 37.9%-F: 62.1% (G2). M: 34.4%-F: 65.6% (G3).	Compare 24 h patterns in medically treated versus **surgically treated** glaucoma patients.	**Surgery Reduces Fluctuation**: The signal fluctuation range was significantly smaller in both surgical groups compared to the medically treated group.
**Huang SK et al., 2023** [32] SENSIMED Triggerfish (mVEq)	Prospective open-label, single-arm study. 6 POAG/NTG subjects. MA: 58.3 ± 13.9 years. M: 66.6%-F: 33.4%.	Compare **circadian IOP** fluctuations before and after **adjunctive ripasudil** eye drop administration.	**Reduced Average IOP**: 24 h average IOP and awake-time average IOP were significantly lower after ripasudil administration (*p* ≤ 0.0265). **Contact Lenses Effect**: CLBS can act as barriers that affect the absorption of eye drops and weaken the IOP-lowering effect.
**Muniesa et al., 2019** [33] SENSIMED Triggerfish (mVEq)	Prospective, nonrandomized case series. 91 subjects with OHM (medical group), and POAG (surgical group). MA: 66.6 ± 8.50 years (OHM). MA: 68.3 ± 12.5 years (POAG). M: 45.8%-F: 54.2% (OHM). M: 59.4%-F: 40.6% (POAG).	**Compare fluctuations** in IOP in medically vs. surgically treated glaucoma patients.	**Fluctuations** related to IOP were greater in eyes with medically treated glaucoma than in those with surgically treated glaucoma.
**Shioya et al., 2020** [34] SENSIMED Triggerfish (vEQq)	Prospective open-label, single-center evaluation. 65 subjects with NTG. MA: 50.8 ± 14.6 (M). MA: 52.6 ± 10.2 years (F). M: 43.1%-F: 56.9%.	Evaluate if CLBS combined with a single GAT reading can predict the **potential for IOP to exceed the diagnostic threshold** (20 mmHg).	**Effectiveness**: Two measurement times (15:00 and 18:00) with 1 tonometry reading provided high sensitivity and high Negative Predictive Value. **Clinical Utility**: CLBS can reduce the number of POAG patients potentially misclassified as NTG without a full 24 h tonometric curve.
**V. Safety, Tolerability, and Adverse Events**			
**Zhang et al., 2022** [35] Pressure-Measuring Contact Lens (mmHg)	Not reported study design. 30 subjects (10 Normal, 20 Glaucoma) for 24 h wear. MA: 24.1 ± 3.4 years (NE). MA: 30.9 ± 9.8 years (POAG). M: 44%-F: 56% (NE). M: 66%-F: 34% (POAG).	Assess the **safety and tolerability** of the novel CLS system.	**Generally Tolerable**: The CLS was found to be potentially safe and tolerable for 24 h monitoring. **Transient Worsening**: Ocular surface indicators (CFS, OSDI, VAS discomfort, TBUT) worsened immediately after measurement but showed significant recovery within one day.
**Otsuka et al., 2020** [36] SENSIMED Triggerfish (mVEq)	Prospective observational study. 56 POAG subjects. MA: 71.2 ± 7.9 years. M: 62.5%-F: 37.5%.	Conduct a **questionnaire survey** on complications and subjective symptoms.	**Common Subjective Symptoms**: Blurred vision (55%), ocular pain (30%), conjunctival hyperemia, and sleeping disorder (29%).
**Morales-Fernandez et al., 2018** [37] SENSIMED Triggerfish (mVEq)	Prospective case–control study. 30 subjects (14 Healthy, 16 Glaucoma). MA: 74,8 ± 6,7 years. M: 42.9%-F: 57.1% (NE). M: 43.8%-F: 66.2% (POAG).	Assess changes in corneal topography and biomechanics after 24 h of CLS wear.	**Topographic Changes**: CLS use caused significant topographic changes (increased steep keratometry, mean keratometry, and astigmatism) after 24 h of wear. **No Biomechanical Change**: Corneal biomechanical properties (CH and CRF) did not significantly change in post-wear.

CCT: Central Corneal Thickness; CFS: Corneal Fluorescein Staining; CH: Corneal Hysteresis; CLBS: Contact Lens Based Sensor; CRF: Corneal Resistance Factor; F: female; GAT: Goldmann Applanation Tonometry; HDT: Head-Down Tilt; HTG: High-Tension Glaucoma patients; IOP: Intraocular Pressure; M: male; MA: mean age; MAPE: Mean Amplitude of Pressure Excursion; mmHg: millimeters of mercury; mVEq: millivolt equivalent; NE: normal eyes; NTG: Normal-Tension Glaucoma patients; OHM: ocular hypotensive medication; OHT: Ocular Hypertension patients; OPA: Ocular Pulse Amplitude; OSAS: Obstructive Sleep Apnea Syndrome; OSDI: Ocular Surface Disease Index; POAG: Primary Open-Angle Glaucoma patients; SD: Standard Deviation; TBUT: Tear Break-Up Time; VAS: Visual Analogue Scale; VF: Visual Field. Bold text is used to emphasize the articles’ authors and key results and background color indicate change of subsection.

**Table 3:** Summary of Performance of EYEMATE-IO and EYEMATE-SC Intraocular Pressure Sensors.

Article & Device	Study Design and Population	Agreement with GAT	Key Findings
**Koutsonas et al., 2018** [38] EYEMATE-IO (1st generation)	Retrospective analysis. 6 patients with POAG. Mean follow-up was 37.5 months. MA: 72.8 ± 2.3 years. M: 33%-F: 67%.	**Uncertain correlation** with GAT.	**Good functionality and tolerability** over time.
**Choritz et al., 2020** [39] EYEMATE-IO (2nd generation)	Prospective study. 22 POAG patients. Follow-up lasted 12 months. MA: 67.8 ± 6.8 years. M: 64%-F: 36%.	**Good agreement** (**ICC** = 0.783) EYEMATE-IO readings 3.2 mmHg higher than GAT (**95% LoA**: −3.8 to 10.2 mmHg).	**Safely implanted and reliable**. This device allows for continual and long-term IOP measurements.
**Micheletti et al., 2025** [40] EYEMATE-IO	Prospective case series. 8 eyes of 8 POAG patients. Mean follow-up was 2.88 years. MA: 70.6 ± 6.7 years. M: 75%-F: 25%.	**MD:** 1.2 mmHg. **95% LoA:** −4.6 mmHg to 7.0 mmHg.	Peak IOP and IOP **fluctuations** from the sensor were significantly **associated with glaucoma progression** (RNFL thinning).
**Szurman et al., 2023** [41] EYEMATE-SC	Prospective study. 24 POAG patients Follow-up was 6 months. MA: 65.1 ± 10.2 years. M: 50%-F: 50%.	MD: 1.31 mmHg (**95% LoA:** −4.92 to 7.55 mmHg). MD: −0.15 mmHg after 6 months (100% within ±5 mmHg).	Sensor was **safe and well-tolerated**.
**Szurman et al., 2023** [42] EYEMATE-SC	Prospective clinical trial. 24 POAG patients. Follow-up 12 months. MA: 65.1 ± 10.2 years. M: 50%-F: 50%.	**MD:** 0.8 mmHg (**95% LoA:** −5.1 to 6.7 mmHg). From 3 months onward, mean difference −0.2 mmHg (**95% LoA:** −4.6 to 4.2 mmHg).	Sensor was **safe and well-tolerated** through 12 months.
**Micheletti et al., 2025** [43] EYEMATE-SC	3-year prospective follow-up study 22 POAG patients. Mean follow-up was 2.7 years. MA: 65.0 ± 10.6 years. M: 53.5%-F: 54.5%.	**MD:** 2.3 mmHg (**95% LoA:** −6.2 to 5.7 mmHg). More than 85% within ±5 mmHg.	**Long-term safety and good tolerability** over 3 years.

F: female; GAT: Goldmann applanation tonometry; IOP: intraocular pressure; LoA: limits of agreement; M: male; MA: mean age; MD: mean difference; POAG: primary open-angle glaucoma; RNFL: retinal nerve fiber layer. Bold text is used to emphasize the articles’ authors and key results.

## Data Availability

No new data were created or analyzed in this study. Data sharing is not applicable to this article.
